# King Edward's Hospital Fund for London Act, 1907

**Published:** 1907-08-03

**Authors:** 


### KING EDWARD'S HOSPITAL FUND FOR LONDON ACT, 1907.

An Act to Incorporate the President and Council of King Edward's Hospital Fund for London; to provide for the Management of the Fund, and for other purposes. Royal Assent, July 26, 1907.

### Preamble.

Whereas His Majesty then Prince of Wales did in the year 1897 invite public subscriptions (annual and otherwise) to a Fund now known as "King Edward's Hospital Fund for London" (hereinafter referred to as "the Fund") with the object of securing more efficient aid and support for Hospitals of London and of thus commemorating the sixtieth anniversary of the reign of Her late Majesty Queen Victoria. And whereas the Fund was established and the same has been generously supported by public subscriptions donations and by legacies and otherwise. And whereas His Royal Highness George Prince of Wales is now President of the Fund. And whereas His Royal Highness has from time to time appointed certain persons to act as a General Council and as Committees and in various other capacities and has with the advice and assistance of such Council and Committees administered the Fund. And whereas it is expedient that for the proper establishment and administration of the Fund upon a permanent basis the President and General Council should be incorporated and that provision should be made for the regulation and management of the Fund and with respect to present and future investments. And whereas the purposes of this Act cannot be attained without the Authority of Parliament. May it therefore please your Majesty that it may be enacted and Be it Enacted by the King's Most Excellent Majesty by and with the advice and consent of the Lords Spiritual and Temporal and Commons in this present Parliament assembled and by the authority of the same as follows (that is to say):—

### Short Title.

1. This Act may be cited as King Edward's Hospital Fund for London Act 1907.

### Incorporation.

2. From and after the passing of this Act His Royal Highness George Prince of Wales now President of the Fund and the present members of the said General Council and the persons who hereafter shall be the President and the Governors and the General Council respectively under this Act are hereby incorporated by the name of "King Edward's Hospital Fund for London" (in this Act referred to as "the Corporation") and by that name shall be a body corporate with perpetual succession and a common seal and with power without any further licence in mortmain to take purchase and hold lands and hereditaments and to sue and be sued and with all other rights powers and privileges of a body corporate.

### Objects.

3. The objects of the Corporation are to administer the King Edward's Hospital Fund for London as existing at the passing of this Act to hold all lands moneys securities and other property real and personal belonging to the Fund to obtain from public benevolence by means of subscription donation bequest or otherwise a continuance of the Fund to invest any moneys so obtained and hold investments of the same to execute any special trusts in connexion with moneys or property held or obtained by the Corporation (not being inconsistent with the purposes of this Act) to apply the capital and the income of the Funds and property of the Corporation or any part thereof subject to any such trusts and to the provisions of this Act in or towards the support benefit or extension of the Hospitals of London or some or any of them (whether for the general or any special purposes of such Hospitals) and to do all such things as may be incidental or conducive to the attainment of the foregoing objects.

### Interpretation.

4. In this Act the expression "Hospitals of London" means and includes the following Institutions namely such present or future Hospitals Convalescent Homes Nursing Homes Nursing Institutions Lying-in Institutions Dispensaries Medical Missions Societies for the provision of Surgical or Medical Aid or Appliances and Institutions for the Rest Relief or Cure of Sick Persons as shall be situate within the County of the City of London or the Metropolitan Police District as existing at the passing of this Act or as the same may be hereafter extended or being situate beyond such County or District shall afford Medical or Surgical treatment or Rest Relief or Cure to Patients all or some of whom are persons usually resident or carrying on their vocation in life within such County or District.

### President.

5. His Royal Highness George Prince of Wales shall during the pleasure of the Sovereign be the President of the Corporation and every succeeding President shall be appointed by and hold office during the pleasure of the Sovereign. Provided that the appointment of every succeeding President shall be made only as follows viz.: If at any time when there shall be no President some person being a son brother or grandson of the Sovereign shall in the opinion of The Lord Chancellor of Great Britain the Prime Minister and the Governor of the Bank of England for the time being be duly qualified to fill the office of President they may recommend the Sovereign to appoint and the Sovereign on such recommendation may appoint such person to be President. In default of the appointment of a President on the occurrence of a vacancy in the office three Governors shall be appointed as herein after mentioned, and all powers belonging to the office of President under this Act shall, during the continuance of such vacancy, be exerciseable by such Governors jointly two of whom shall be a quorum and all powers of the President and General Council under this Act shall be exerciseable by such Governors and the General Council. The Governors shall be appointed from time to time by the Sovereign on the recommendation of the Lord Chancellor of Great Britain the Prime Minister and the Governor of the Bank of England for the time being or a majority of them and shall hold office for five years or until the appointment of a President and shall be eligible for re-appointment.

### Powers of President and General Council.

6. (1) The direction and management of the affairs of the Corporation and the administration and distribution of the property and income thereof shall subject to the provisions of this Act be vested in the President and General Council. (2) The Members of the General Council shall from time to time be appointed by the President as he may think fit so however that the number of Councillors so far as may be practicable shall not be less than twelve. All Councillors and Members of Committees constituted under this Act shall hold office until the end of the year in which they shall have been appointed and may be reappointed from year to year. (3) At any meeting of the President and General Council at which the President is present if there is a difference of opinion on any question other than a question as to which a majority of Members of the General Council is required by sub-Section (4) of this section the determination of the President shall be final and all acts done at a meeting of the President and General Council in the absence of the President shall require the approval in writing of the President. Provided that this sub-section shall not apply to questions as to the objects or the manner for or in which property or income of the Corporation is to be distributed dealt with or applied. (4) A majority of the Members of the General Council present at a Meeting of the President and General Council may from time to time by resolution subject to the approval of the President delegate the power of the General Council with respect to any matters within the powers of the President and General Council to any Committee constituted under this Act and thereupon the President and any such Committee shall with respect to those matters exercise the powers of the President and General Council and sub-section (3) of this section shall apply to meetings of the President and any such Committee as if the same were meetings of the President and General Council.

### Special Powers of President.

7. The following powers shall subject to the provisions of this Act be vested in the President (that is to say)—

(1) He may from time to time by writing under his hand constitute Committees for the administration of the Funds and property of the Corporation or for any of the purposes of this Act and appoint the members thereof and may delegate to any Committee all or any of his powers under this Act as he may think fit including the power of approval in writing given by sub-Section (3) of the Section of this Act the heading of which is "Powers of President and General Council" which power if delegated to a Committee of two persons shall be deemed to be sufficiently exercised by each of such persons signing an approval in writing of the act approval whereof is required. (2) He may appoint and remove any Treasurers Secretaries Auditors and other Officers of the Corporation and fix their remuneration. (3) He may determine whether any institution does or does not fall within the definition of "Hospitals of London" in this Act contained.

### Regulations, Procedure, etc.

8. Subject to the provisions of this Act the President and General Council may make rules and regulations for the management and administration of the Fund and for the holding of meetings and procedure and the conduct of the business of the Corporation including the use of the Corporate seal and the keeping of regular books and accounts.

The accounts of the Fund shall be annually audited by an auditor nominated by the President of the Local Government Board. The remuneration of such auditor shall be fixed by the President of the Local Government Board and paid out of the Fund.

Transfer of Existing Property and as to Property Generally.

9. All real and personal property now belonging to or held by any person or persons in trust for the Fund shall forthwith after the passing of this Act be assured transferred delivered and paid by such person or persons to or otherwise vested in or placed under the control of the Corporation but as to property given to the Fund upon any special trust or condition now affecting the same subject to such trust or condition. All gifts by will or otherwise which if this Act had not been passed would have taken effect in favour of the Fund shall enure for the benefit of the Corporation.

All property of the Corporation for the time being not applied for the purposes thereof shall be vested in or placed under the control of the Corporation.

Investment of Funds.

10. (1) All moneys and funds of the Corporation which are not immediately required to be expended for the purposes thereof and which the Corporation think proper to be invested shall be placed in such investments as may be authorised with respect thereto by or by the powers contained in the instrument (if any) of gift of such moneys or funds or of the moneys or property from which the same shall have arisen or by the powers contained in any writing or writings under the hand of the donor according to the provisions of this Section or so far as such instrument writing or writings do not extend in investments authorised by the law for the time being in force for the investment of Trust Funds. Provided that the Corporation shall not invest money in or retain any securities in respect whereof any liability exists unless the liability is of limited amount and is to be discharged or is capable if the Corporation think fit of being discharged within a fixed period from the date of investment but save as in this proviso mentioned nothing in this Act shall prevent the Corporation from the full exercise of any discretion or authority given by the donor in the choice of investments. All investments may be varied or transposed from time to time into or for other investments authorised according to the provisions of this Section with respect to the original investments or the proceeds thereof. For the purposes of this Section land shall be deemed to be an investment. (2) Where at the passing of this Act the Fund comprises money or other property real or personal which has been given by a donor or is the proceeds or an investment of a donor's gift or where after the passing of this Act any money or other property is given by a donor to the Corporation either generally or in a special trust any such donor may (within six months after the passing of this Act or six months after the gift as the case may be) by writing or writings under his hand confer on the Corporation any such powers or additional powers of investment reinvestment sale retention or otherwise with respect to his gift and the income thereof as he may think fit and any such writing or writings shall have effect as if the powers had been contained in an instrument of gift of such money or other property to the Corporation. (3) Land and hereditaments or any interest therein vested in or belonging to the Corporation may be sold mortgaged charged or leased for any period as if the Corporation were a beneficial owner. The provisions of Section 5 of the Mortmain Act 1891 as to the sale of land assured by Will to or for the benefit of a charitable use shall not apply to gifts by Will to the Corporation. (4) Moneys of the Corporation awaiting distribution or investment may be either deposited with the Bankers of the Corporation or advanced on the security of stocks funds or securities the purchase of which would be authorised by the law for the time being in force for the investment of trust funds. (5) Any stocks funds or securities forming part of the Fund at the passing of this Act and the retention whereof shall not be authorised under the provisions of this Section shall be realised within twelve months from the passing of this Act.

Gifts Subject to Conditions.

11. The Corporation may accept gifts and endowments of real and personal estate either for special purposes of the Corporation or in aid of the general purposes thereof and upon such terms and conditions (not inconsistent with the objects of the Corporation) as may be agreed upon between the Corporation and the person bestowing such gifts and endowments.

Indemnity.

12. All persons hitherto acting in the management and application of the Fund shall be indemnified by virtue of this Act in respect of all acts and things done by them or any of them in the execution or intended execution in good faith of the trusts of the Fund as heretofore constituted.

Costs of Act.

13. The costs charges and expenses preliminary and of and incidental to preparing and obtaining this Act shall be paid by the Corporation.

